# The FreeD module’s lateral translation timing in the gait robot Lokomat: a manual adaptation is necessary

**DOI:** 10.1186/s12984-023-01227-3

**Published:** 2023-08-18

**Authors:** Tabea Aurich (-Schuler), Florian van Dellen, Rob Labruyère

**Affiliations:** 1 Children’s Research Center, University Children’s Hospital Zurich, Steinwiesstrasse 75, Zurich, CH-8032 Switzerland; 2 Swiss Children’s Rehab, University Children’s Hospital Zurich, Mühlebergstrasse 104, Affoltern am Albis, CH-8910 Switzerland; 3 Sensory-Motor Systems Lab, Department of Health Sciences and Technology, ETH Zurich, Tannenstrasse 1, Zurich, CH-8092 Switzerland

**Keywords:** Robot-assisted gait therapy, Trunk trajectory, Center of mass displacement, Weight shifting, Pelvis movements, Rehabilitation, Marker-less motion system

## Abstract

**Background:**

Pelvic and trunk movements are often restricted in stationary robotic gait trainers. The optional FreeD module of the driven gait orthosis Lokomat offers a combined, guided lateral translation and transverse rotation of the pelvis and may therefore support weight shifting during walking. However, from clinical experience, it seems that the default setting of this timing does not correspond well with the timing of the physiological pelvic movement during the gait cycle. In the software, a manual adaptation of the lateral translation’s timing with respect to the gait cycle is possible. The aim of this study was to investigate if such an offset is indeed present and if a manual adaptation by the therapist can improve the timing towards a more physiological pattern comparable to physiological overground walking.

**Methods & Results:**

Children and adolescents with neurologic gait disorders and a Gross Motor Function Classification System level I-IV completed two different walking conditions (*FreeD Default* and *FreeD Time Offset*) in the Lokomat. The medio-lateral center of mass positions were calculated from RGB-Depth video recordings with a marker-less motion capture algorithm. Data of 22 patients (mean age: 12 ± 3 years) were analyzed. Kinematic analyses showed that in the *FreeD Default* condition, the maximum lateral center of mass excursion occurred too early. In the *FreeD Time Offset* condition, the manual adaptation by the therapists led to a delay of the maximum center of mass displacement by 8.2% in the first phase of the gait cycle and by 4.9% in the second phase of the gait cycle compared to the *FreeD Default* condition. The maximum lateral center of mass excursion was closer to that during physiological overground walking in the *FreeD Time Offset* condition than in the *FreeD Default* condition.

**Conclusion:**

A manual adaptation of the timing of the FreeD module in the Lokomat shifts pelvis kinematics in a direction of physiological overground walking. We recommend therapists to use this FreeD Time Offset function to adjust the phase of weight shifting for each patient individually to optimize the kinematic walking pattern when a restorative therapy approach is adopted.

**Supplementary Information:**

The online version contains supplementary material available at 10.1186/s12984-023-01227-3.

## Background

Walking short distances was found to be the most frequently mentioned mobility goal of children and adolescents with neurological diagnoses undergoing rehabilitation [1]. Besides that, a gait pattern similar to that of typically developing children is considered a treatment outcome of high importance in youth with neurologic gait impairments and their parents [2]. For a stable human gait, center of mass (COM) movement over the stance leg is crucial to avoid falls. This is achieved by a lateral translation of the pelvis and the trunk, which involves a natural medial shift of the knee and relative hip adduction during the stance phase [3, 4]. The first maximum lateral excursion of the COM occurs at around 30% of the gait cycle (GC), during the end of early midstance phase when the COM is shifted over the straight stance limb. The second maximum towards the other side follows approximately at 80% of the GC towards the midstance phase of the other leg [5].

In rehabilitation, besides conventional physical therapy, stationary robotic gait trainers such as the Lokomat (Hocoma AG, a DIH brand, Volketswil, Switzerland) are applied to train or maintain the patients’ ability to walk. However, such devices usually fixate the pelvis, and a study with healthy adults has shown that this fixation during treadmill walking distorts gait kinematics [6]. Furthermore, pelvic restrictions are also suspected to influence the muscle activation patterns when walking in a robotic gait therapy device on a treadmill with limited degrees of freedom in the frontal plane [7]. For the Lokomat, a commercially available add-on has been recently released to tackle this issue. The FreeD module is an optional hard- and software-related technology that guides the pelvis on a semi-elliptical path, providing a coupled lateral translation and transverse rotation. The extent of the lateral translation and the timing of the maximum pelvic excursion in relation to the GC can be individually adjusted (the setting is called “FreeD Time Offset”), and it must be activated in the software settings screen (see supplemental material 1a). Additionally, the leg cuffs can be released to passively follow the medio-lateral displacement of the pelvis to a certain degree. Therefore, the FreeD module should support weight shifting during walking in the Lokomat (see [8] for more details).

In clinical practice, however, therapists observe that the timing of maximum pelvic excursion in the frontal plane with the FreeD default settings tends to occur too early in the GC, namely around loading response rather than the midstance phase.

In this study, we used a marker-less motion tracking system to compare the timing of the COM displacement between two different approaches: (I) During walking in the Lokomat with default FreeD settings (*FreeD Default)*, and (II) during walking in the Lokomat with a patient-individualized time offset chosen by the therapist (*FreeD Time Offset*). We hypothesized that the maximum lateral displacement of the COM in the *FreeD Default* condition occurs earlier and that an individualized timing offset shifts the maximum lateral displacement of the COM closer to a physiological point in the GC.

## Methods

### Participants

Participants were in- and out-patients of the Swiss Children’s Rehab of the University Children’s Hospital Zurich, Switzerland, all showing neurologic gait impairments. Participants between 5 and 18 years of age were recruited by convenience sampling.

For eligibility, patients had to meet the following inclusion criteria: (1) Gross Motor Function Classification System (GMFCS, [9]) levels I-IV (I: walks without limitations, IV: severely limited self-mobility even with assistive technology, mostly transported in a wheelchair), (2) prior experience of at least three Lokomat therapies, (3) ability to understand simple instructions and to express pain or discomfort, (4) fulfillment of the criteria described in the user manual for the Lokomat [10], (5) written informed consent.

The participants were characterized with the GMFCS (if diagnosed with cerebral palsy (CP)) and the Trunk Control Measurement Scale (TCMS, [11]).

### Experimental design

This study was approved by the Cantonal Ethics Committee of Zurich (BASEC-Nr. 2019–02116) and conducted following the Declaration of Helsinki. All the measurements were performed at the Swiss Children’s Rehab of the University Children’s Hospital Zurich in Affoltern am Albis, Switzerland. Information about the study was presented orally to the participants and their legal guardians in advance. Participants younger than 14 years were included upon verbal assent, while participants aged 14 or older had to provide additional written consent to participate in the study. Written informed consent by a legal guardian was required.

### Devices and outcome measures

#### Lokomat

Detailed information about the Lokomat Pro (Version 6, Hocoma AG, Volketswil) and the FreeD module can be found elsewhere [8, 10]. The Lokomat orthoses were adjusted individually to each participant according to clinical standards and expert experience. During an accommodation period of 10 min, Lokomat parameters, such as the hip and knee joint range of motion, were individually adjusted for the participant. The speed was gradually increased until the participant rated the walking pace as comfortable. The Lokomat guidance force was set to 100% to prevent compensatory movements in the upper body that would interfere with the effects of the FreeD module. Bodyweight support was initially set to 30% and then reduced as much as possible while still ensuring a stable and safe walking pattern with adequate knee extension during the stance phase. Participants were instructed to loosely place their hands on the parallel bars and look straight ahead to minimize contributions from voluntary arm and head movements during walking.

This study compared two different approaches: *FreeD Default* and *FreeD Time Offset*. In both conditions, the pelvis was actively guided through a lateral excursion of 2 cm to each side and a coupled transversal rotation of around 4° per side. Moreover, to facilitate medio-lateral weight shifting over the stance leg, the upper and middle leg cuffs at the thighs and upper shanks were released (in our customized Lokomat adult orthosis: maximal medio-lateral translation of 1.5 cm to each side; Lokomat pediatric orthosis: maximal medio-lateral translation of 1 cm to each side, see supplemental material 1b for further explanation). In the *FreeD Default* condition, the time offset was set to 0%, which corresponds to the default setting in the software (see supplemental material 1c for further explanation). During the *FreeD Time Offset* condition, therapists manually adjusted the timing of the maximum pelvic excursion. The timing can be adjusted in the software from − 10% (earlier) to + 10% (delayed) in steps of 1% of the GC (see supplemental material 1c for further explanation). Therapists were instructed to adjust the timing such that the maximum lateral pelvic excursion towards the stance leg corresponded to the midstance phase, just before the contralateral leg was brought forward to the swing phase [5]. This manual adaptation based on the visual assessment of the therapists. Three different therapists performed the measurements, and all were very experienced in pediatric Lokomat therapy and gait analysis (8.2 ± 4.3 years of experience (mean ± standard deviation (SD)).

The order of the two walking conditions was randomized. Participants could familiarize themselves with each walking condition for 1–2 min, then followed the 3 min recording time. Between the conditions, there was a 2 min break in a neutral standing position. The instructions given before and during the measurement were standardized and can be found in the supplemental material (see supplemental material 2).

#### RGB-Depth recording

Data for the marker-less motion tracking was obtained with a single RGB-Depth camera (Azure Kinect DK, Microsoft, Seattle, USA) at a sampling rate of 30 Hz and was placed 1.5 m in front of the participant walking in the Lokomat.

### Data recording and processing

The frontal video recordings of the gait pattern were processed offline with a method previously validated by van Dellen et al. [12]. In short, RGB-D data were transformed to three-dimensional point clouds. Scale, shape, and pose parameters of a Sparse Trained Articulated Human Body Regressor (STAR, [13]) were optimized based on the point clouds. The resulting model provides kinematic data as three-dimensional time series for 24 joints. For this analysis, only medio-lateral COM positions were analyzed. To this end, data were transformed from the camera coordinates into a patient coordinate frame defined by the floor plane and the walking direction. The time series were then segmented into individual strides and time normalized (0-100% of the gait cycle). Twenty-five steps were processed and analyzed at the end of the 3 min recording time, which should provide sufficient time for motor adaptation beforehand [14].

### Data analysis and statistics

Matlab (Version R2021b, the Mathworks, Natick, USA) was used to calculate the COM trajectories for each participant and condition. Using the crosscorrelation function, we calculated the time delay 𝛕 of the COM trajectory of the *FreeD Time Offset* condition compared to the COM trajectory of the *FreeD Default* condition for each participant. Furthermore, the time points of the absolute minimum (COM excursion to the left) and maximum (COM excursion to the right) in the time normalized gait cycle were calculated for each participant for both conditions. Then, 𝛕 and differences of the time points of the minima and maxima between the conditions were averaged per group. SPSS (Version 27, IBM Corporation, Armonk, USA) was used to test the data for normal distribution (Kolmogorov-Smirnov test) and to perform inferential statistics with an alpha level of 0.05. We used the one-sided t-test to determine if 𝛕 was larger than zero. We examined the similarity between 𝛕 and the offset set by the therapists with the Spearman’s correlation coefficient and interpreted it as follows (adopted from Evans [15]): r < 0.20, “very weak”; 0.20–0.39, “weak”; 0.40–0.59, “moderate”; 0.60–0.79, “strong” and 0.80–1.00 “very strong relationship”. We used the paired-samples t-test to determine differences between the minima and maxima of the trajectories between *FreeD Default* and *FreeD Time Offset*.

## Results

### Participants

In total, 25 children and adolescents could be recruited. The main characteristics of the participants and Lokomat related parameters are summarized in Table 1. Two participants had to be excluded due to non-compliance and equipment malfunctioning. One participant had to be excluded due to poor data quality. The remaining 22 patients (8 girls, 14 boys, 12 ± 3 years (mean ± SD)) walked in the Lokomat with a mean velocity of 2.0 ± 0.4 km/h and mean bodyweight support of 17 ± 8%.

**Table 1:** Participants’ characteristics and Lokomat related parameters

ID	age	Diagnosis	GMFCS	TCMS	Lokomat orthosis	BWS (%)	Gait speed (km/h)	Offset (% GC)
1	10	Spastic movement disorder of unclear aetiology	no	33	P	15	2	NA
2	11	Bilateral spastic CP	IV	14	P	27	1.5	1
3	14	Bilateral spastic CP	III	21	P	13	2.1	6
4	12	Bilateral spastic CP	II	38	A	12	2.4	6
5	12	Bilateral spastic CP	II	39	A	29	2	6
6	12	Bilateral spastic CP	IV	8	P	30	2	4
7	17	ABI	no	46	A	7	3.2	5
8	11	Bilateral ataxic CP	I	43	A	19	2.1	5
9	12	Bilateral spastic CP	II	37	A	13	2.2	7
10	16	ABI	no	43	A	11	2.6	4
11	11	ABI	no	48	P	10	1.9	3
12	11	ABI	no	50	A	18	2.1	6
13	8	Neuromuscular movement disorder after infection	no	58	P	16	2.1	6
14	9	Bilateral spastic CP	III	28	P	21	1.8	4
15	12	MMC	no	29*	P	16	1.6	5
16	10	ABI	no	38	P	11	2	7
17	14	Bilateral spastic CP	I	44	A	10	2.6	6
18	14	Bilateral spastic CP	III	12	A	37	1.3	2
19	15	Bilateral spastic CP	II	48	A	9	1.9	6
20	7	MMC	no	30	P	26	1.7	5
21	15	MMC	no	48	A	21	2.2	7
22	14	ABI	no	46	A	7	2.3	NA
**mean ± SD**	12 ± 3			37 ± 14		17 ± 8	2 ± 0.4	5 ± 2

Legend: ID = identification number; GMFCS = Gross Motor Function Classification System; TCMS = Trunk Control Measurement Scale; BWS = Bodyweight Support in % of bodyweight; Gait speed of the Lokomat treadmill in km/h; Offset for *FreeD Time Offset* in % of GC; GC = gait cycle; CP = cerebral palsy; ABI = acquired brain injury; MMC = meningomyelocele; no = not applicable (the GMFCS is not validated for diagnoses other than CP); P = Pediatric Lokomat orthosis; A = Adult Lokomat Orthosis; NA = not available (missing data); *= TCMS items 8, 9, 10 and 12 not tested due to spinal fusion T2-L5. SD = Standard Deviation.

### Center of mass displacement within a gait cycle

**Fig. 1:**
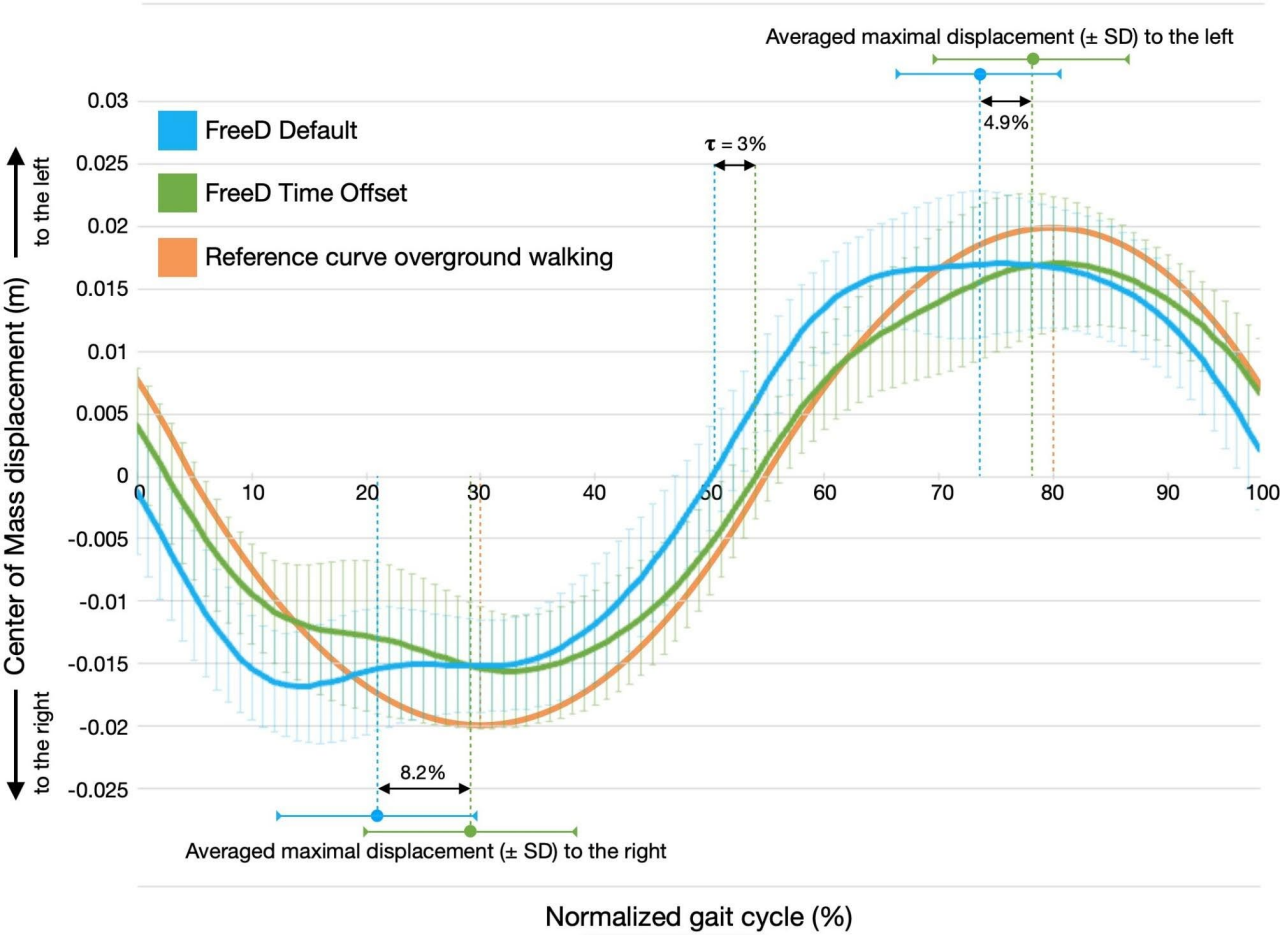
Averaged medio-lateral COM displacement across the gait cycle for the two FreeD conditions. Legend: Two different Lokomat FreeD conditions (*FreeD Default* in blue and *FreeD Time Offset* individually selected by therapist in green) are shown across the gait cycle (0-100%). A reference curve of the COM during overground walking is presented in orange (adopted from [5]). Note: The curves are the averaged COM trajectories over all participants; the data of the averaged maximal displacement, however, stem from individualized calculations and where only then averaged. Accordingly, the green and blue dashed lines are not exactly on the local minima and maxima of the displayed averaged curves. The COM displacements are depicted as mean ± SD in meters. COM = center of mass; SD = standard deviation

Figure 1 presents the participants’ averaged medio-lateral COM displacement over a full GC (right heelstrike to the right heelstrike) of both conditions. The mean time offset of the *FreeD Time Offset* condition determined via crosscorrelation was 𝛕 = 3 ± 1.8% (p < 0.01) in the positive (= delayed) direction of the GC compared to the *FreeD Default* condition.

The peak excursion of the COM to the right over the averaged GC of all participants shifted from 20.9% (*FreeD Default*) to 29.1% (*FreeD Time Offset*, p < 0.01, see Fig. 1). The peak of the maximal translation of the COM to the left shifted from 73.4% (*FreeD Default*) to 78.3% (*FreeD Time Offset*, p < 0.01, see Fig. 1) of the GC.

Therapists chose a mean time offset in the software of 5 ± 2% also in a positive (= delayed) direction during the *FreeD Time Offset* condition compared to the default setting. The Spearman correlation coefficient between the offset chosen by the therapist and 𝛕 was ρ = 0.60, p < 0.01.

## Discussion

In this study, we investigated two different approaches of the Lokomat FreeD module influencing the timing of the lateral pelvis translation in relation to the GC. In the *FreeD Time Offset* condition, therapists on average delayed the timing of maximal pelvic excursion of the FreeD module by 5 ± 2% (mean ± SD). This resulted in a delay of the peak lateral center of mass (COM) excursion of 8.2% in the first part of the GC and 4.9% in the second part. Over the entire gait cycle, the crosscorrelation analysis revealed an averaged offset difference of 3 ± 1.8%. Figure 1 also shows that the COM trajectory during the *FreeD Time Offset* condition is very similar to the reference trajectory from overground walking (adopted from [5]).

A proof of concept for the FreeD has already been described by Aurich-Schuler et al. [16]. The authors reported that walking with FreeD led to a significantly larger pelvis displacement in healthy adults compared to walking with a fixed pelvis [16]. However, in clinical routine, therapists observed that maximum pelvic excursion during the *FreeD Default* condition occurs earlier, namely at a time after loading response instead of midstance. According to literature, the maximum medio-lateral displacement of the COM occurs at approximately 30% and 80% of the gait cycle and correspond to the maximum weight shift over the right and the left standing leg [5].

In the current study, we were able to show that during the *FreeD Time Offset* condition, the peaks of the medio-lateral COM excursion fit the timing described in the literature quite accurately (29.1% of GC and 78.3% of GC), whereas the *FreeD Default* setting occurred too early, especially in the first part of the GC (20.9% of GC, 73.4% of GC). This indicates that a patient-individually adapted time offset of the Lokomat FreeD by the therapist, can shift the COM displacement in a direction that is more similar to physiological walking than with the default setting.

Furthermore, the selected offset and the measured offset correlated significantly but only moderately. The moderate correlation here could have different reasons. The attachment of the participants’ belt to the Lokomat allows for a certain degree of kinematic freedom, which could be responsible for a difference in the selected and the measured offset. Furthermore, our analysis investigated the COM (inside the body), whereas the therapists obviously look at the body’s surface to estimate the time offset. We selected the COM because our kinematic model based on the STAR allows for a more precise and reliable estimation of the COM compared to a reference point on the surface of the model, which can be subject to distortions of the point cloud. While the movements of the COM and the pelvis during Lokomat walking are closely linked, small differences between both perspective frames could occur. Along the same line, therapists adjust the time offset visually by looking at the maximum lateral pelvic excursion and not to the COM. In the other hand, the therapist always look at the whole gait pattern to aim for a physiological walking pattern. Thereby, the therapist automatically includes additional movements of the patient (e.g., from the upper body, shoulders, etc.) in his decision to adapt parameters.

Based on the findings of this study, we want to encourage therapists to use the *FreeD Time Offset* option to facilitate the best possible physiological weight shifting and COM displacement. This report emphasizes that current technology does not replace therapists, who continue to play a key role in robot-assisted therapy. Therapists’ experience and knowledge are crucial tailoring Lokomat parameters to patients’ skills and therapy goals. This cross-sectional study does not make statements on differences in training effectiveness of both approaches regarding patients’ walking abilities, however, it can help therapists to understand the immediate effects of the device’s technical options and selectively apply these to patients. This, in turn, can help define ideal treatment protocols to plan further clinical trials.

### Limitations

This study has some limitations. First, we solely focused on COM kinematics and did not assess leg or trunk kinematics, which may also be affected by the FreeD time offset function. However, we aimed to compare the *FreeD Default* and the *FreeD Time Offset* conditions as straightforward as possible to show whether there is a detectable difference between these approaches and whether they should be considered in the clinic and for future research.

Secondly, our study showed, that the manual adaptation of the FreeD’s timing led to a more physiologic gait pattern. However, its cross-sectional study design did not allow to evaluate, whether the more physiological gait pattern also led to a more effective Lokomat training. Future studies should investigate, whether approximating the gait pattern in the Lokomat to a physiological overground pattern also has a clinical impact on the effectiveness of this form of therapy.

### Practical considerations

Several considerations regarding the Lokomat hardware and software settings have to be made. The attachment of the participant’s belt to the Lokomat in the pediatric leg orthoses allows for more kinematic freedom compared to the adult leg orthoses. It may therefore be that less time offset is required for pediatric Lokomat orthoses since the child has more freedom to “override” the actuated lateral displacement of the FreeD module itself.

Additionally, it should be mentioned that our customized FreeD system at the Swiss Children’s Rehab allows the cuffs to shift medially and laterally (see supplemental material 1b). In our opinion, this is the right approach to not only allow the knee to slide inward during the guided lateral pelvic translation (which could in our opinion favor an excessive, relative valgus position), but also to allow weight shifting, where the thigh and the knee can slide laterally. Furthermore, FreeD software settings were set to 2 cm lateral pelvis translation to both sides for all participants (according to the literature [3]), regardless of their height and leg length. It is unclear whether individually adjusted settings would have influenced the results. The time offset adaptation by the therapist (-10% to + 10%) cannot be made based on single percentage points of the gait cycle. Accordingly, the authors suggest converting this scale (e.g., “small” to “strong” time offset) in a future software update.

A further weakness of the FreeD module is that it is actuated. Consequently, patients do not have to actively control the translation and rotation of the pelvis in the device, which can lead to them not doing so at all - a phenomenon named “slacking” [17].

Further, experienced therapists observe that the required *FreeD Time Offset* increases with faster walking speeds. A study by Schwarz et al. showed that the kinematics, kinetics, spatio-temporal parameters, and surface electromyography of typically developing children change when gait speed changes [18]. Therefore, the time offset should be adjusted if the gait speed is varied during the therapy. Even better would be an automatic coupling of these parameters. However, this study did not systematically investigate how the kinematic parameters or the shape of the trajectory change when the gait speed changes, thus, further Lokomat FreeD studies should address this topic.

Finally, it is important to note that the goals of therapy do not have to be focused exclusively on “normal gait mechanics” or on walking as physiologically as possible (restorative approach of robot-assisted therapy). Likewise, therapy goals based on “trial and error” or compensatory directions also have their justification. With our investigation, we do not want to evaluate whether one of the two is more favorably but emphasize again the importance of the therapist to decide which approach is preferable in the patient-tailored and goal-oriented therapy.

## Conclusion

This study showed that the maximum lateral COM displacement occurs too early in the GC when the default FreeD time offset is used. Therefore, a manual adaptation of this setting by the therapist led to a shift of the maximum lateral COM displacement towards the pattern seen during physiological overground walking, where it occurs during the early midstance phase, just before the contralateral is brought forward to the swing phase. We, therefore, encourage Lokomat therapists to consider the immediate effects of this technical option and apply them in a patient-tailored approach.

### Electronic supplementary material

Below is the link to the electronic supplementary material.

Supplemental Material 1 (a-c): Notes about the FreeD Settings.

Supplemental Material 2: Standardized test instructions.

Supplemental Material 3: STROBE Statement checklist.

Supplemental Material 4: Source data.

## Data Availability

The datasets supporting the conclusions of this article are included in the supplemental material (see supplemental material 4).

## Abbreviations

ABI: acquired brain injury
COM: center of Mass
CP: cerebral palsy
GC: gait cycle
GMFCS: Gross Motor Function Classification System
MMC: myelomeningocele
RGB-D: Red Green Blue-Depth
SD: standard deviation
STAR: Sparse Trained Articulated Human Body Regressor
TCMS: Trunk Control Measurement Scale
