# Supplementary material for: The FreeD module’s lateral translation timing in the gait robot Lokomat: a manual adaptation is necessary
Source: J Neuroeng Rehabil. 2023 Aug 18;20:109. doi: 10.1186/s12984-023-01227-3 (PMC10439589; doi:10.1186/s12984-023-01227-3)

**Supplemental Material 1 (a-c): Notes about the FreeD Settings**

(a) FreeD activation in the Lokomat software settings screen (Lokomat User Manual L6.2-UM6.5-en, page 230):


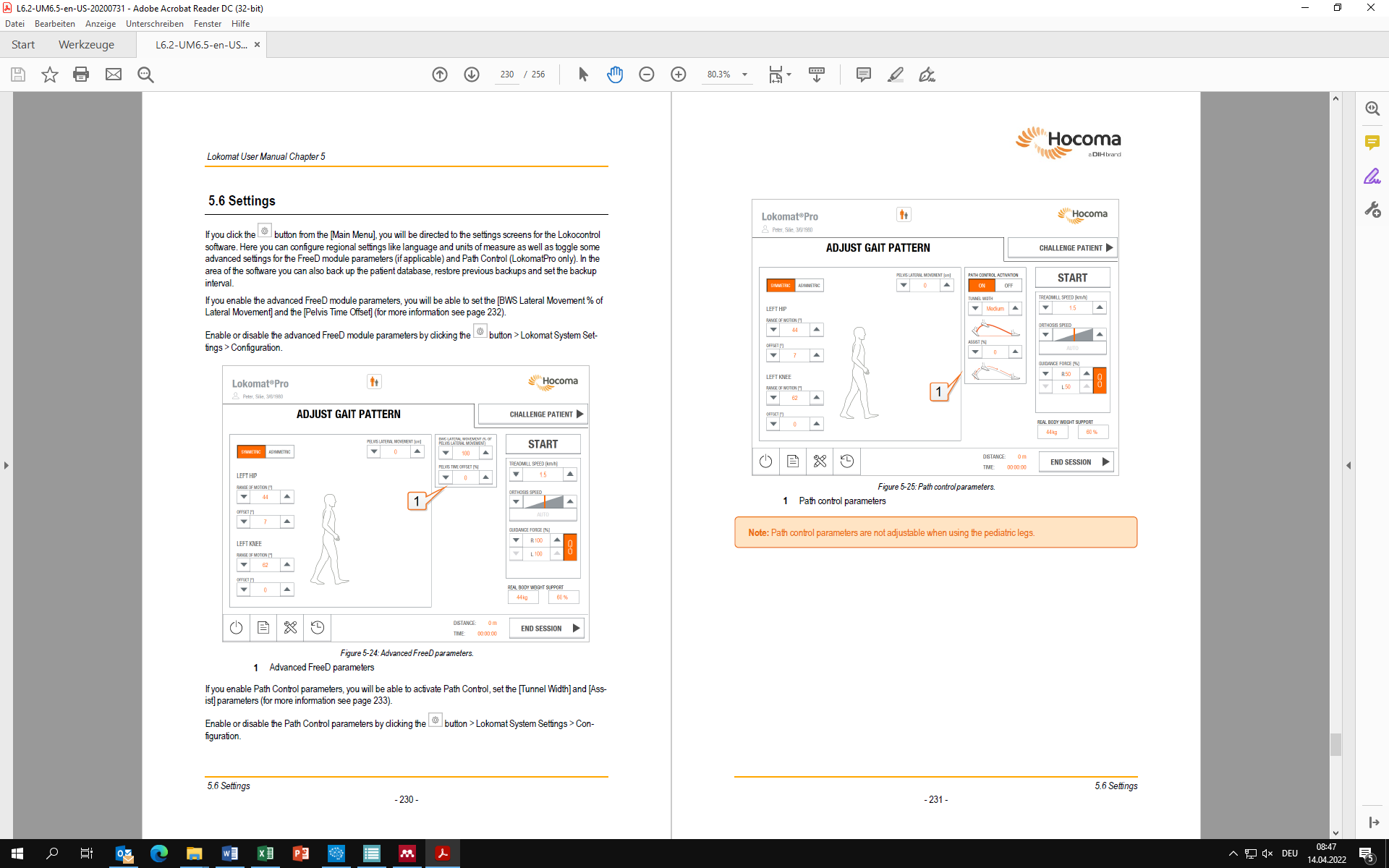


(b) Lokomat FreeD cuffs (Lokomat User Manual L6.2-UM6.5-en, page 175): Conventional FreeD system: Cuff can only be fixed at one point (lateral) and can therefore only slide inward=medial.


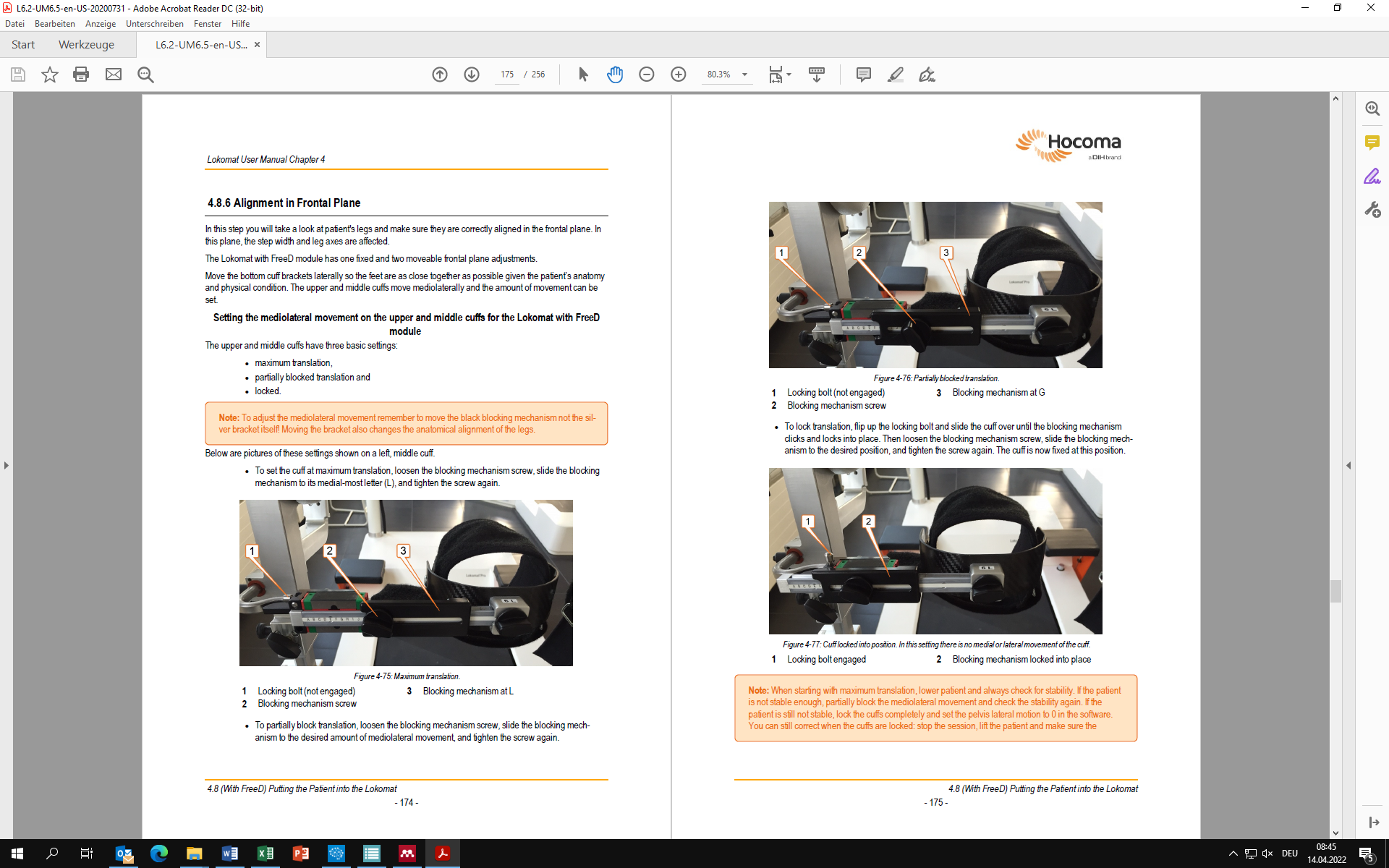


Our FreeD system has 3 fixation points (lateral, middle, medial) and the cuff can slide from the middle point (our default setting) to the outside/lateral and to the inside/medial direction.


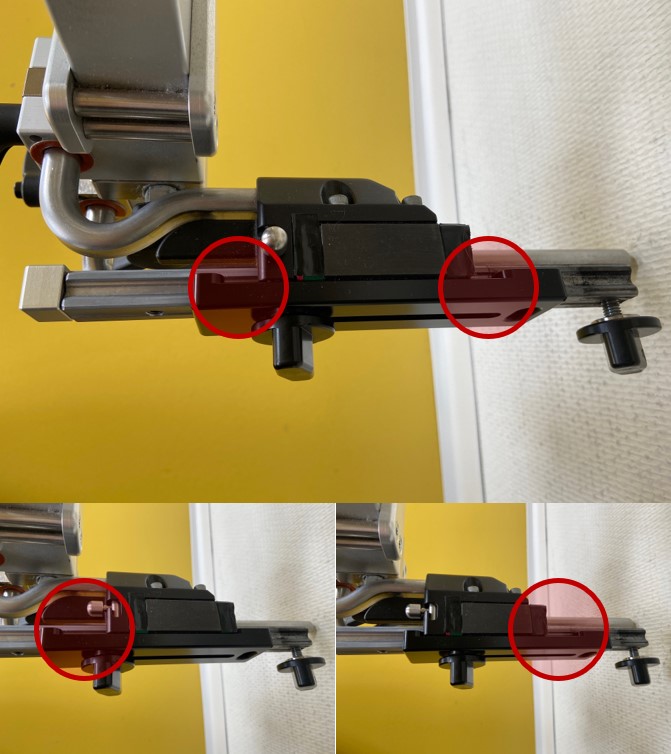


(c) Advanced FreeD Parameters explanation (Lokomat User Manual L6.2-UM6.5-en, page 232):


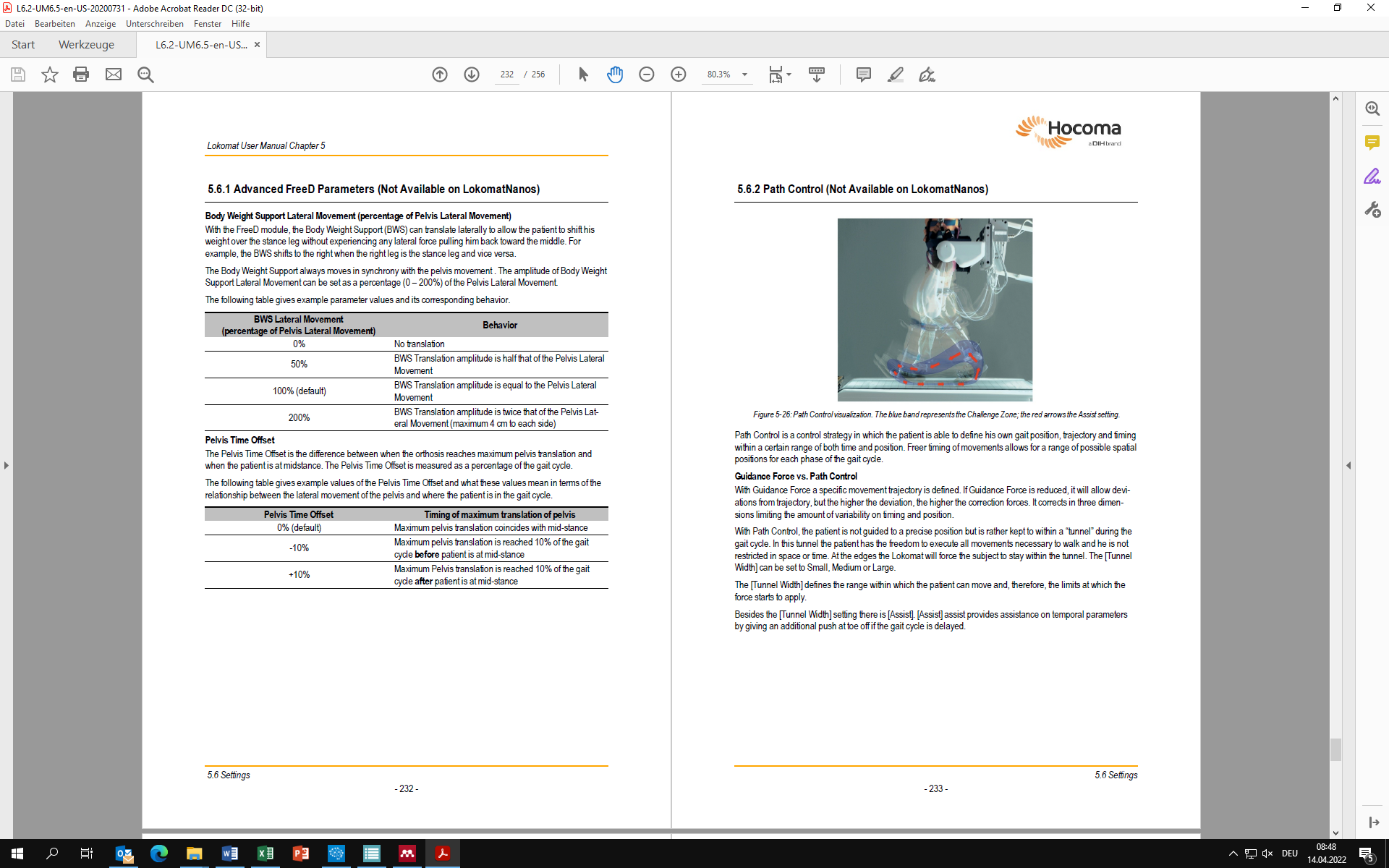

Supplement: Supplementary file 1 — Supplemental Material 1 (a-c): Notes about the FreeD Settings. [file 12984_2023_1227_MOESM1_ESM.docx]
